# Feasibility of A-mode ultrasound based intraoperative registration in computer-aided orthopedic surgery: A simulation and experimental study

**DOI:** 10.1371/journal.pone.0199136

**Published:** 2018-06-13

**Authors:** Kenan Niu, Jasper Homminga, Victor I. Sluiter, André Sprengers, Nico Verdonschot

**Affiliations:** 1 Laboratory of Biomechanical Engineering, Faculty of Engineering Technology, MIRA Institute, University of Twente, Enschede, the Netherlands; 2 Orthopaedic Research Lab, Radboud University Medical Center, Nijmegen, the Netherlands; Universite Catholique de Louvain, BELGIUM

## Abstract

**Purpose:**

A fast and accurate intraoperative registration method is important for Computer-Aided Orthopedic Surgery (CAOS). A-mode ultrasound (US) is able to acquire bone surface data in a non-invasive manner. To utilize A-mode US in CAOS, a suitable registration algorithm is necessary with a small number of registration points and the presence of measurement errors. Therefore, we investigated the effects of (1) the number of registration points and (2) the Ultrasound Point Localization Error (UPLE) on the overall registration accuracy.

**Methods:**

We proposed a new registration method (ICP-PS), including the Iterative Closest Points (ICP) algorithm and a Perturbation Search algorithm. This method enables to avoid getting stuck in the local minimum of ICP iterations and to find the adjacent global minimum. This registration method was subsequently validated in a numerical simulation and a cadaveric experiment using a 3D-tracked A-mode US system.

**Results:**

The results showed that ICP-PS outperformed the standard ICP algorithm. The registration accuracy improved with the addition of ultrasound registration points. In the numerical simulation, for 25 sample points with zero UPLE, the averaged registration error of ICP-PS reached 0.25 mm, while 1.71 mm for ICP, decreasing by 85.38%. In the cadaver experiment, using 25 registration points, ICP-PS achieved an RMSE of 2.81 mm relative to 5.84 mm for the ICP, decreasing by 51.88%.

**Conclusions:**

The simulation approach provided a well-defined framework for estimating the necessary number of ultrasound registration points and acceptable level of UPLE for a given required level of accuracy for intraoperative registration in CAOS. ICP-PS method is suitable for A-mode US based intraoperative registration. This study would facilitate the application of A-mode US probe in registering the point cloud to a known shape model, which also has the potential for accurately estimating bone position and orientation for skeletal motion tracking and surgical navigation.

## Introduction

Computer-Aided Orthopedic Surgery (CAOS) systems have been developed, validated and used for surgeries in the lower extremity, such as total knee arthroplasty (TKA) [1, 2] and total hip arthroplasty (THA) [3]. CAOS systems offer several advantages over traditional surgery: improving guidance of the surgical instruments, reducing complication rates, minimizing trauma from instrument access and allowing preview and measurement of anatomical regions in a virtual environment [4–6]. In some of CAOS scenarios, medical images of a patient are acquired preoperatively, for example from Computed Tomography (CT) or Magnetic Resonance Imaging (MRI), and used to plan the surgical steps. During surgery, the preoperative image data then need to be registered to the actual patient. The first step is to acquire intraoperative data (e.g. digitized points, lines, curves or surfaces) from the anatomy of the actual patient in the operating room. The second step is to use an appropriate registration algorithm to determine the transformation that matches the preoperative data to intraoperative data.

The acquisition of intraoperative data can be done by using various types of markers such as adhesive skin markers [7, 8], anatomical landmarks and implantable bone markers, all of which have advantages and disadvantages. Skin markers are non-invasive, but the skin movements relative to the bone may dramatically decrease the accuracy of registration [8]. Anatomical landmarks on specific locations of patient’s anatomy can be detected and digitized utilizing pointer probes (tracked by an optical or electromagnetic navigation system) [9], but using anatomical landmarks often requires surgical exposure of additional bony surfaces, causing additional trauma and extension of the operating procedure. Implanted bone markers provide a high registration accuracy, and are commonly considered as the gold standard [10]. The implanted markers can be tracked using intraoperative fluoroscopy or CT [11]. In cadaver experiments, the error has been reported to be 0.99 ± 0.41mm [12]. However, implanted markers have the drawback of exposing the patient to additional radiation and trauma, because it is necessary to affix the implant markers to the patient before pre-operative scanning.

To avoid most of aforementioned drawbacks, ultrasound (US) offers a non-invasive and non-radiative approach for intraoperative registration. As ultrasound is capable of detecting the bone boundaries through the soft tissue, ultrasound-based intraoperative registration eliminates the need for physical contact with the bone surface [13]. A-mode (Amplitude modulation, a display of amplitude of received echo as a function of depth through a single transducer scanning) ultrasound has already been used for intraoperative registration [14–17]. Compared to the conventional B-mode (Brightness-mode, a display of 2D image in which image intensity depends on the amplitude of received B-mode echoes through an array of transducers) ultrasound transducers, A-mode ultrasound transducers are cheaper and easier to determine the bone surface in real-time [18, 19], as only 1-dimensional signal needs to be processed. Besides, the size of A-mode ultrasound transducer is suitable to be attached to different anatomical areas. Its localization accuracy was reported to be approximately 0.4 mm after calibration [14, 16, 20]. Since the calibration procedure is indispensable, it would need an additional procedure before intraoperative registration. The combination of a CAOS-system and single A-mode ultrasound transducer has been used in skull surgery [21, 22], pelvis surgery [23], and knee surgery [16]. Hence, to utilize A-mode ultrasound transducers with its inherent localization error for intraoperative registration, a clearly defined registration procedure should be established. Different from the image registration methods that are normally used in B-mode ultrasound based registration, A-mode based registration typically uses a 3D point cloud obtained by the transducer to register on a known shape model of the bony segment. The Iterative Closest Points (ICP) algorithm is commonly used to compute the transformation between the point cloud and shape model [24]. Generally, a larger number of points will probably lead to a more accurate registration, but herein a tradeoff lies between registration accuracy and the time spent on point acquisition. In this study, the combination of ICP with a Perturbation Search algorithm was presented for A-mode ultrasound based intraoperative registration with less registration points than conventional ICP would require [16, 25].

Furthermore, the registration accuracy will not only depend on the number of points acquired, but also on the errors to measured points (termed Ultrasound Point Localization Error, UPLE). To assess the sensitivity of these aspects in a systematic manner, *in-vivo* or *in-vitro* experiment would not be the first option as it is very difficult to change one factor while keeping the others constant. We therefore first performed a numerical simulation (i.e. Monte-Carlo simulation) to investigate the effects of two aspects on the registration accuracies of our method and the standard ICP: (1) the number of ultrasound registration points and (2) UPLE. Subsequently, a cadaveric experiment was conducted to verify the simulation outcomes and to validate the proposed registration method in practice.

## Materials and methods

### 2.1 Data generation for simulation

In this study, we focused on the femur bone as object of study. A shape model (a STL file format, contains 37745 vertices) of a healthy femoral bone was generated from CT data in the previous study [26]. This model was considered as a known shape model that represents the preoperative data of the patient (termed the Pre-Operative Model, POM). It was used to generate the ultrasound registration points.

**Fig 1:**
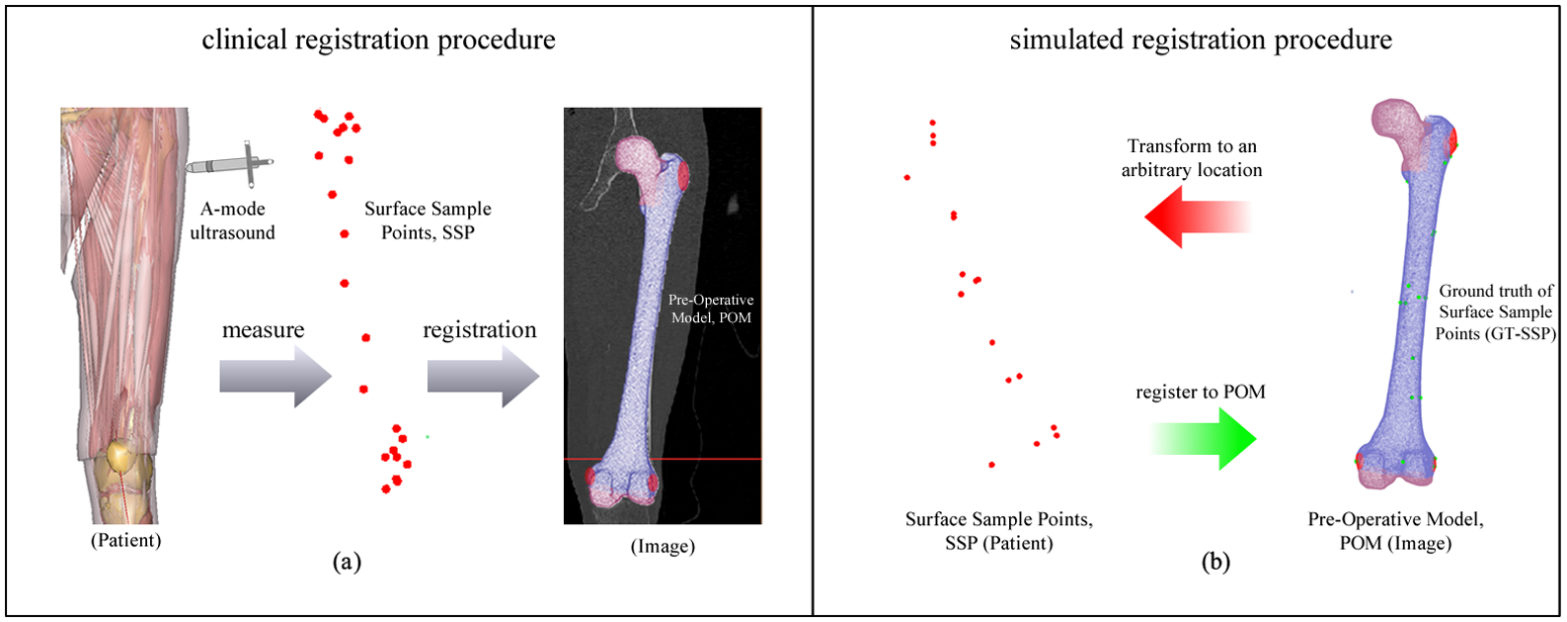
The comparison between the clinical registration procedure and simulated registration procedure. (a) the illustration of clinical registration procedure including measuring ultrasound points from patient and registering them to the pre-operative model. (b) the illustration of simulated registration procedure involving generating the ultrasound point from pre-operative model and transforming them to a new position rigidly, eventually registering those points back to the pre-operative model.

In the clinical registration procedure, the measured points on the bony surface of the actual patient via A-mode ultrasound (termed the Surface Sample Points, SSP) can thus be registered to the POM, as shown in Fig 1a.

**Fig 2:**
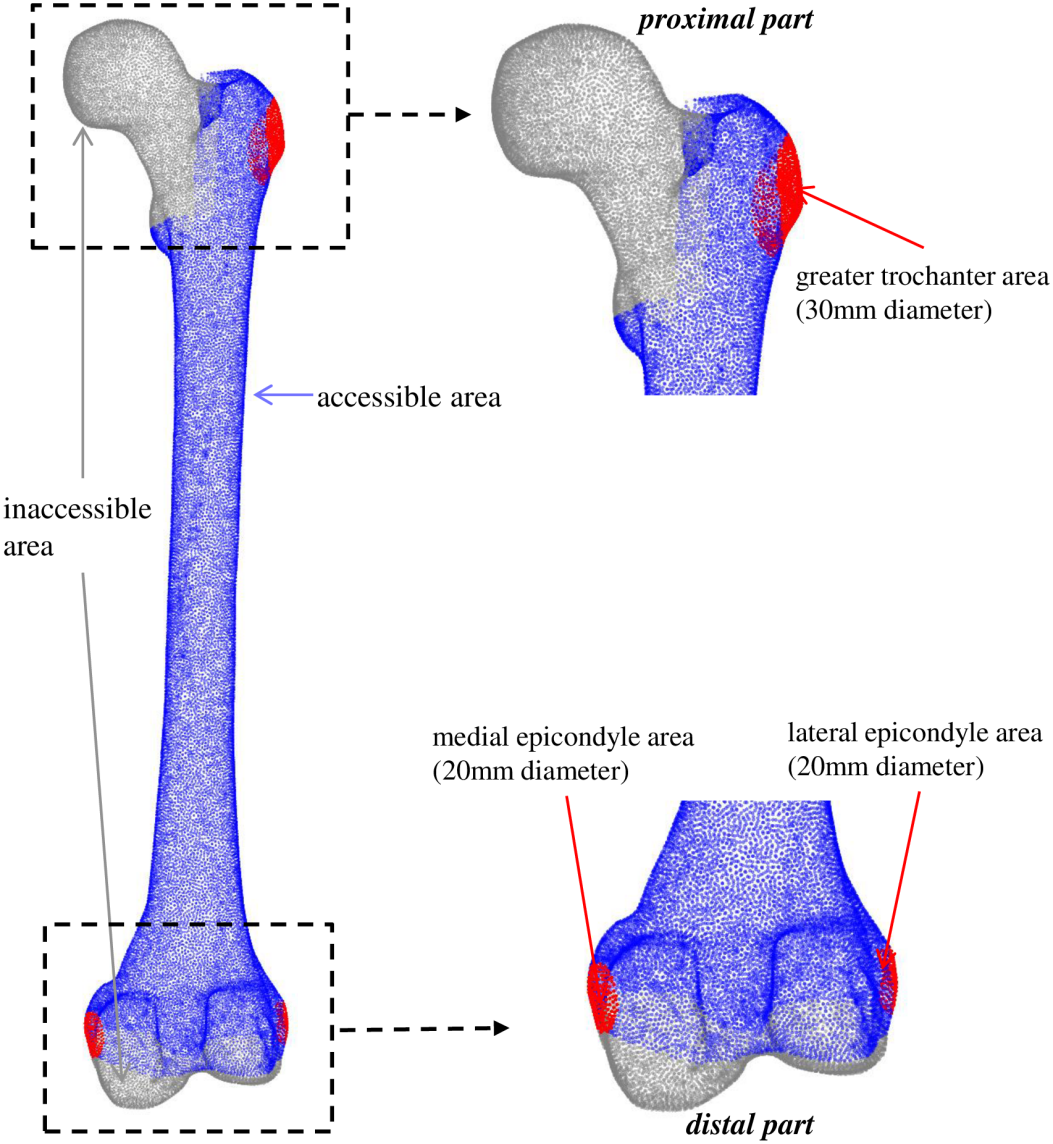
The inaccessible areas (gray), accessible areas (blue) and pre-registration areas (red) on POM. The pre-registration areas include: the greater trochanter area (30mm diameter), medial epicondyle area (20mm diameter) and lateral epicondyle area (20mm diameter); shaft areas around the middle shaft of the femur: lateral, medial, anterior, and posterior (20 diameters for each side).

The simulated registration procedure started with the acquisition of a set of points from the POM. A set of points was selected from all areas that were accessible to A-mode ultrasound [16] as shown in Fig 2. The spatial locations of these selected points were served as ground truth for assessing the registration accuracy (termed GT-SSP). The corresponding SSP were generated by transforming the GT-SSP to an arbitrary new location, resulting in a set of points being seen as ultrasound sample points in the clinical registration procedure. The selection of GT-SSP was performed by the following two-step protocol:

1. The first six points of the GT-SSP were picked from three pre-defined restricted areas, termed pre-registration areas. These areas were easy to locate and detect in the clinical scenario. To simulate different conditions that would happen in practice, two points were randomly selected from each pre-registration area (Fig 2): two points from the greater trochanter area, two points from the medial epicondyle area and two points from the lateral epicondyle area. These six points were used for pre-registration of the SSP to the POM, which will be discussed later.
2. The rest of the GT-SSP were randomly picked from all accessible areas (as shown in Fig 2).

### 2.2 Registration method

After generating GT-SSP and corresponding SSP, the registration method (ICP-PS) was performed in a four-step procedure: Firstly, a point-to-point preregistration was performed, using the 6 points from the pre-registration areas. Secondly, the Iterative Closest Point (ICP) algorithm was applied, using all of the points from the SSP to register to the POM. Thirdly, to avoid local minima in the ICP, a systematic perturbation was applied to explore if there were better registration results available compared to the first ICP result, based on the Point-to-Surface Euclidean Distance (PSD) between the registered SSP and the POM. Fourthly, the feedback loop including perturbation procedure and ICP was then repeated until the convergence of the PSD was reached. Each of these steps was described in more detail in the following sections.

#### 2.2.1 Pre-registration: 1^st^ step

The six points measured from the pre-registration areas were used for pre-registration (coarse registration). Point-to-point rigid registration was utilized to fit the six points to a set of corresponding points locating at the centroids of the pre-registration areas. Point-to-Point rigid registration is a process to find the adequate transformation between target points and measured points[27]. The objective function is defined as:

$$f(R,T)=\frac{1}{n}\sum_{i=1}^{n}{∥x_{i}-(Ru_{i}+T)∥}^{2}$$ (1)

where *U* = {*u*_*i*_} is a set of first *n* points of SSP (*n* = 6 in our simulation) and *X* = {*x*_*i*_} is the set of all the corresponding points on POM, which are centroids of pre-registration areas, ***T*** represents the translation vector and ***R*** represents the rotation matrix between the SSP and the POM.

#### 2.2.2 Iterative Closest Point (ICP): 2^nd^ step

The maximum number of iterations of the ICP algorithm was set to 30 in this study. The details of ICP algorithm can be found in [24]. For each iteration, firstly, the closest points on the POM with respect to the SSP were calculated to establish the correspondence point pairs. Secondly, a point-to-point registration was applied on the correspondence point pairs to get an updated SSP. Then the first and second steps were repeated until meeting ending conditions (beyond the iteration times or convergence). To speed up the ICP algorithm, k-d tree [28] was used for searching the closest points of the SSP on the POM, which was the most time consuming computation in the ICP algorithm.

#### 2.2.3 Perturbation search: 3^rd^ step

**Fig 3:**
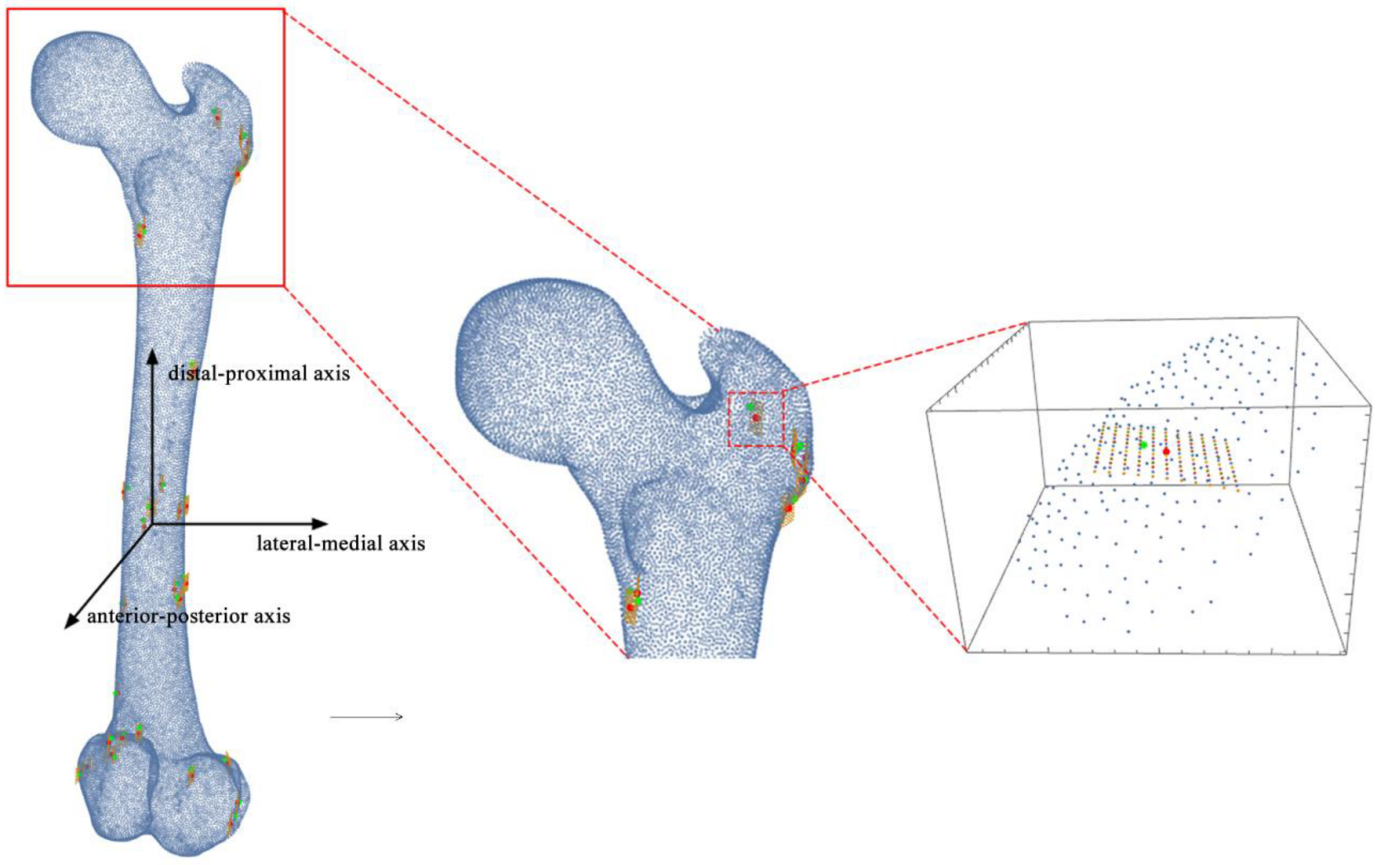
The illustration of Perturbation search. The curved grid of perturbation searches with 143 combinations of rotations and translations for each point. Green point represents the perturbed sample points. Red point represents the registered points of ICP.

ICP and its variants are local optimization methods, which may get stuck in local minima of the objective functions. It is difficult to find the global minimum from an arbitrary starting position without the pre-registration process [29]. Our method was inspired by the method of Ma and Ellis [29]. The registered SSP were perturbed rigidly from its ICP registered position and the new perturbed position was verified whether it represented a smaller local minimum. Perturbation can obviously be done in all directions. Instead of perturbing points randomly, we, based on a pilot study, assumed that most of mismatching occurred along the distal-proximal axis of the femur rather than the anterior-posterior axis or the lateral-medial axis (Fig 3). The reason for this also is the fact that the femur has a cylinder-like shape. When the number of ultrasound registration points is too small to provide strong geometrical constraints in all directions (missing points from the proximal and distal parts of the femur), it is more likely to occur translational and rotational misalignments around the distal-proximal axis of the femur. The perturbations were therefore implemented as rotations around the femoral-distal-proximal axis from -5 to 5 degrees with intervals of 1 degree and translations along the same axis from -3 to 3 mm with intervals of 0.5 mm. The perturbations can thus be seen as a curved grid around the bone of 143 (11 by 13) combinations of rotations and translations (Fig 3). For each perturbation in this curved grid, the Point-to-Surface Euclidean Distance (PSD) was calculated and compared to the PSD of the original ICP result:

$$PSD=\frac{1}{n}\sum_{i=1}^{n}{∥s_{i}-u_{i}∥}^{2}$$ (2)

where *S* = {*s*_*i*_} is a set of *n* closest points on the POM surface. Each *s*_*i*_ is calculated by each *u*_*i*_, and *U* = {*u*_*i*_} is perturbed sample points.

#### 2.2.4 Feedback from perturbation to ICP: 4^th^ step

If the perturbation procedure produced a more optimal result than the ICP result from the second step (section 2.2.2), the ICP algorithm was applied again from the perturbed position. The feedback loop including perturbation and ICP was then repeated until the convergence of the PSD was reached, i.e. PSD_n+1_ –PSD_n_ <0.00001, with a maximum of 5 repetitions.

#### 2.2.5 Definition of registration accuracy

The accuracy of the registration was estimated by calculating the root mean square error (RMSE) of Euclidean distances between the registered POM (*U* = {*u*_*i*_}) and the known ground truth of the POM (*G* = {*g*_*i*_}), where *n* represents the number of points in the POM. Therefore, the RMSE calculation represents a bone-to-bone error metric:

$$RMSE=\sqrt{\frac{1}{n}\sum_{i=1}^{n}{{\left(\|u_{i}-g_{i}\right\|}^{2})}^{2}}$$ (3)

### 2.3 Simulation procedure

In the simulation phase, the accuracy of the registration method was assessed as a function of the number of registration points for different levels of UPLE. To compare our method with the standard ICP, the registration accuracies were calculated at different registration steps (after the second, third and fourth step of the registration procedure). The number of registration points ranged from 6 to 25 (i.e. 20 different sizes for registration points). The UPLE may stem from several sources, e.g. calibration error (caused by calibration procedure approximate 0.4 mm) [16], speed of sound that would affect the depth calculation (*vt*/2; where *v* is the velocity of sound in the material (1590 m/s in muscle across the fibers [30] and *t* represents the time that ultrasound waveform takes from the origin of ultrasound beam to the bone surface and reflect back to the origin) [31], navigation system (intrinsic error of navigation system). An investigation of the noise model of SSP was beyond the scope of this study, hence, we assumed the noise had isotropic distribution in all direction and was added to SSP randomly. The UPLE was simulated as a noise vector in random direction with the magnitude uniformly distributed over a preset interval[32]. The interval was varied from 0 (i.e. no UPLE), [0–1] and [1–2] mm. We chose this range based on previous studies that reported errors varied from 0.5 to 2mm [16, 20, 22]. The procedures from generating SSP and GT-SSP to applying registration on SSP were repeated one hundred times. In total, we thus ran 6000 (20 x 3 x 100) simulations.

The simulation program was implemented in Mathematica (Wolfram Mathematica 10.3.0). The hardware configuration of computer was Intel Core i7-4800MQ (2.70GHz) and 8G RAM. The time efficiency of a single simulation (including from Select GT-SSP to whole registration procedure) was 11.08 seconds on average. The total time cost of all simulations was about 18 hours.

### 2.4 Cadaver experiment

In this study we used one human cadaveric specimen. Testing on cadaveric knees is necessary to ensure the safety and functioning of the developed technology before we apply this to patients. At the Radboud University Medical Center (Radboud UMC) we have a long history of performing cadaveric experiments and we established working principles to ensure all ethical issues and legal aspects are covered. Radboud UMC has an Anatomical department which has the authority under Dutch law to use human tissue for educational and research purposes. The Dean of the Medical faculty is responsible for the ethical issues with regard to the use of human cadaver material. The cadaveric experiment was conducted according to the protocol issued by the department of anatomy of the Radboud UMC and were approved by both our local ethics advisor, Dr. Frans Huysmans (Internist-nephrologist, Concernstaf Quality and Safety, Radboud University Medical Center) and the European Research Council (UMCRPS02PRD_PRTC0119). None of the transplant donors were from a vulnerable population and all donors or next of kin provided written informed consent that was freely given.

**Fig 4:**
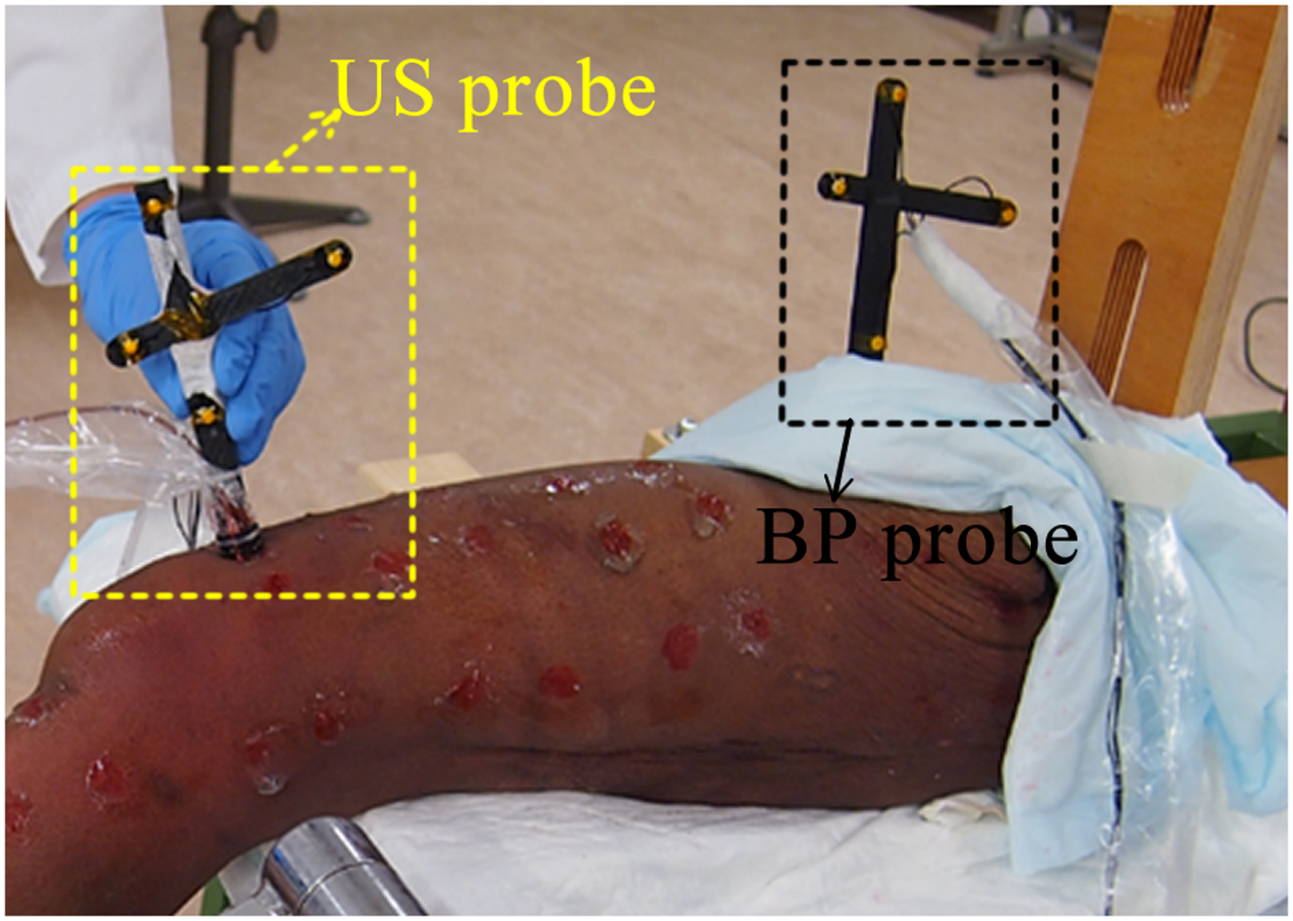
The setup of cadaver experiment and ultrasound probe and bone pin probe. Both the ultrasound probe and bone pin probe have four optical markers. The bone pin probe provides the ground truth position of the underlying bone.

To verify the outcomes of the simulation and to assess the performance of our method in practice, a cadaveric experiment was conducted on a left leg. Prior to CT scan, one Bone Pin probe (BP probe) was screwed onto the proximal part of femur, with a frame containing four optical markers (Fig 4). Then a CT scan was made at the department of Radiology of the Radboud UMC using TOSHIBA Aquilion ONE (TOSHIBA, Tustin, USA) with voxel size of 0.755 mm × 0.755 mm × 0.500 mm. The image was segmented manually in Mimics^®^ 17.0 (Materialise N.V., Leuven, Belgium) and the surface model of the femur was generated for registration. The 3D locations of four optical markers of the BP probe were also manually digitized from the CT data. The femoral head was fixed in a custom setup and the leg was kept stationary during experiment, as shown in Fig 4. One Ultrasound probe (US probe) that contained four optical markers and one 7.5 MHz A-mode ultrasound transducer (Imasonic SAS, Voray / l’Ognon, France) was used to acquire ultrasound sample points. An optical tracking system (Visualeyez VZ4000v trackers, PTI Phoenix Technologies Inc., Vancouver, Canada) was operated at 100 Hz to track the 3D locations of BP probe and US probe. The ultrasound signal was acquired and synchronized with the optical tracking system in the Diagnostic Sonar FI Toolbox (Diagnostic Sonar Ltd., Livingston, Scotland). Custom written software was developed to process all data in LabVIEW 2014 (National Instruments, Austin, USA). The origin and direction of ultrasound beam were determined from the calibration method [16]. The ultrasound echo signal was filtered and a peak detection window was manually set to find the maximum peak and thus to determine the bone depth. Subsequently, the ultrasound sample point could be digitalized in 3D space [20].

The protocol to define the locations where points should be collected was similar to the protocol in the simulated registration procedure (section 2.1). Instead of randomly acquiring points, we measured points from 25 anatomical areas including pre-registration areas, which were distributed homogeneously and covered anterior, lateral and medial parts of femur. For each anatomical area, the US probe acquisition was repeated 10 times. After acquiring all points, the UPLE of each point was estimated by calculating the PSD between each point and the ground truth location of the femur derived by the BP probe. To simplify the permutation of all acquired points, only the maximal PSD sample point (i.e. sample point was located farthest from bone) and the minimal PSD sample point (point was closest to bone) out of 10 measurement points were selected for each anatomical area. Then, the selected points was sorted in the descending order for both maximal and minimal point sets. Subsequently, registration was performed for 6 points increasing to 25 points in descending order of UPLE for both the maximal and minimal case, which guaranteed that the registration accuracy would increase when adding sample points. In the rest of this study, these two datasets will be referred to as “closest” and “farthest”. Both standard ICP and ICP-PS method were applied onto these two datasets. The registration accuracies of these two registration methods were calculated by the bone-to-bone error metric as same as in the simulation.

## Results

### 3.1 Simulation results

**Fig 5:**
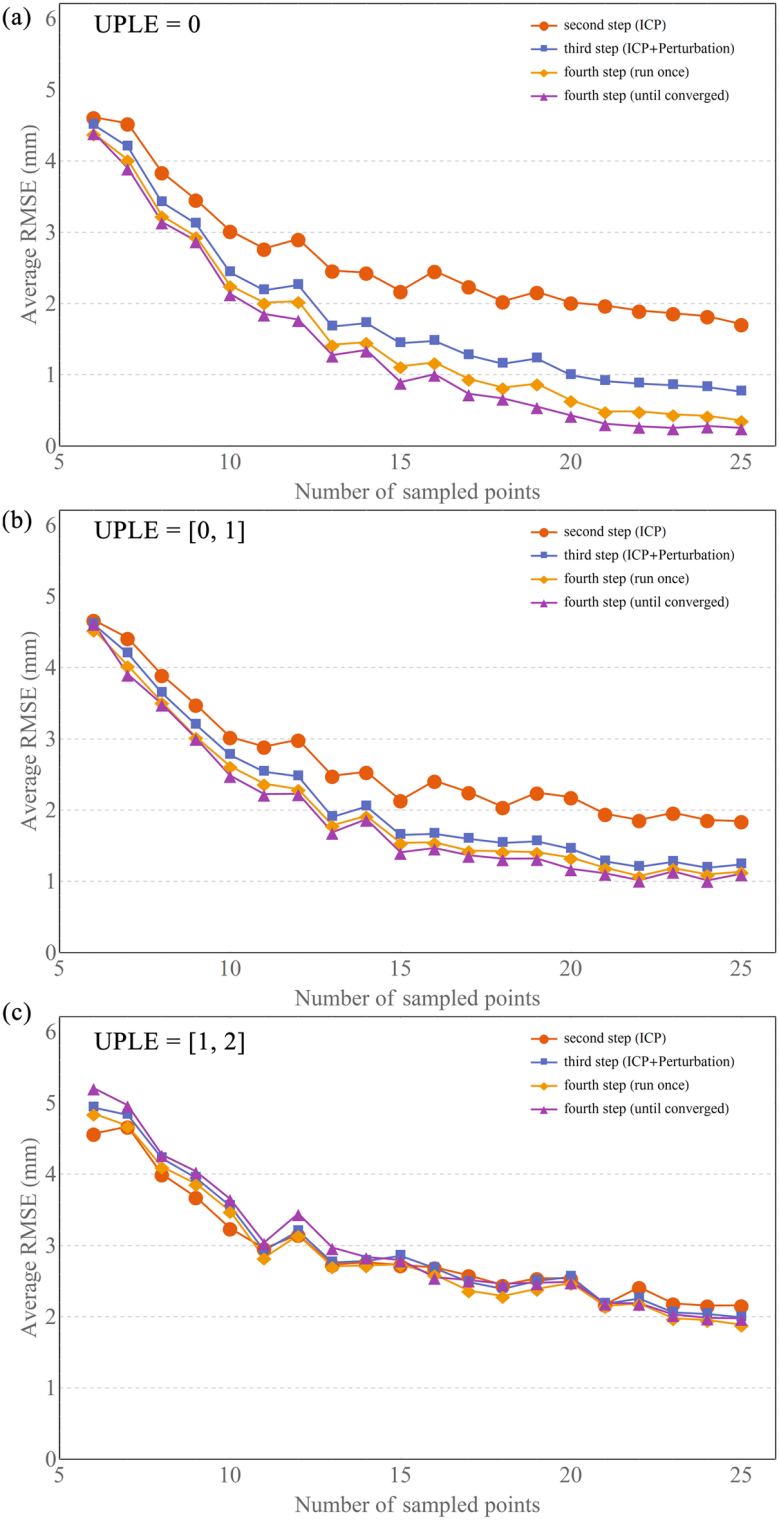
The simulation results of average RMSE after different registration steps. (a) the average RMSE after different registration steps with the UPLE set to zero. (b) the average RMSE after different registration steps with the UPLE set to [0, 1]. (c) the average RMSE after different registration steps with the UPLE set to [1, 2].

The registration accuracy (average RMSE) as a function of the number of sample points for different registration procedures was shown in Fig 5. When the UPLE set to zero, the final registration error of ICP method (the second step) reached 1.71 mm for 25 sample points (Fig 5a). After the third step (perturbation search), this error dropped to 0.76 mm (Fig 5a). The fourth step, (i.e. repeating ICP and perturbation search until convergence was reached) yielded a further drop to 0.25 mm (Fig 5a). The registration error of ICP-PS decreased by 85.38%, compared to that of ICP. The presence of UPLE at the sample points clearly reduced the accuracy of the registration. The improvement of registration accuracy from the second step to the fourth step became less effective with higher UPLE (Fig 5a and 5c).

Generally, the registration accuracies of both ICP and ICP-PS improved by increasing the number of sample points for all three UPLE levels. With a non-zero UPLE, the average RMSE still decreased with increasing the number of points, but the rate of descent declined (Fig 5).

### 3.2 Cadaver experiment results

**Fig 6:**
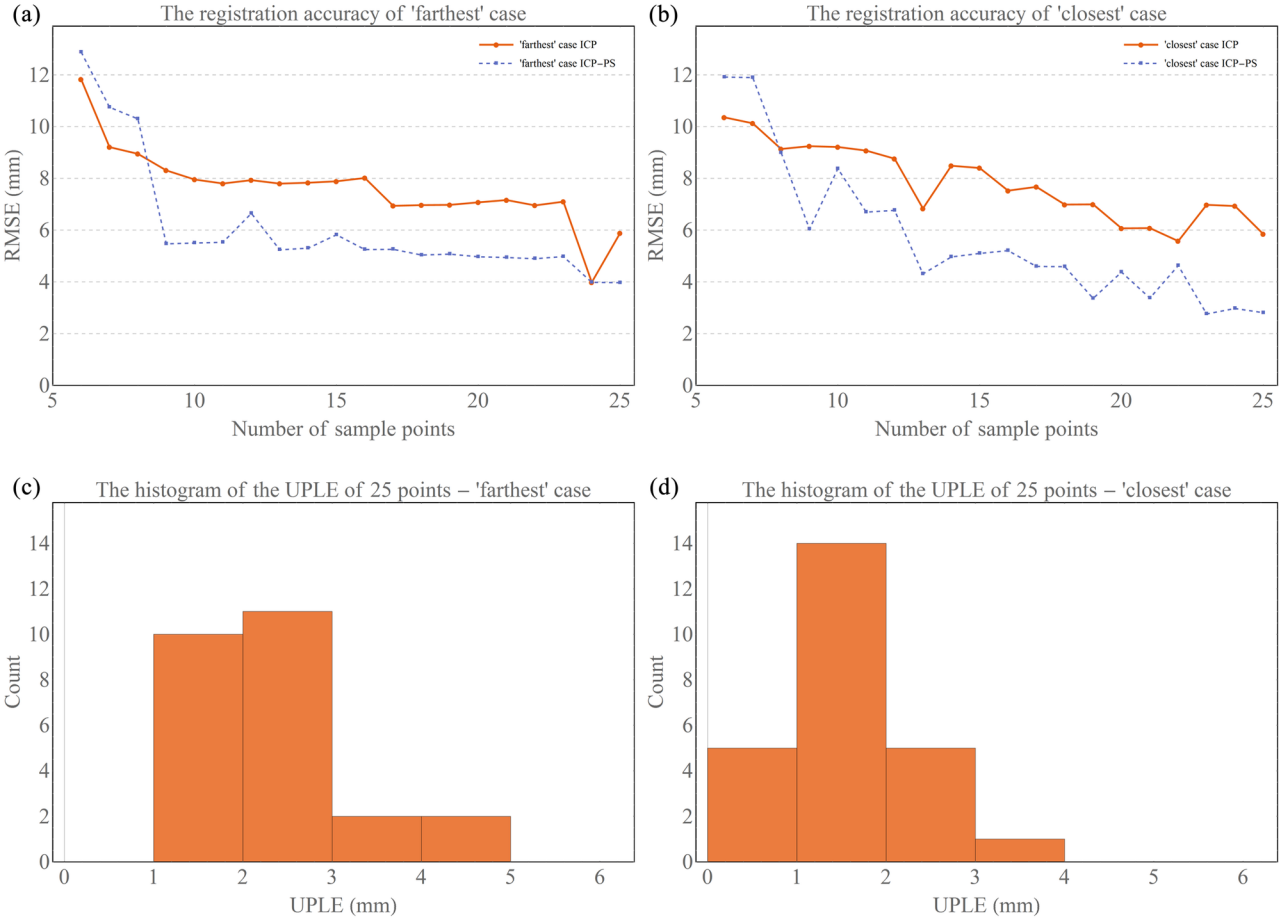
The cadaver experiment results of registration accuracy and UPLE. (a) The registration accuracy of different registration methods on the “farthest” case. (b) The registration accuracy of different registration methods on the “closest” case. (c) the histogram of the UPLE of 25 point for the “farthest” case. (d) the histogram of the UPLE of 25 points for the “closest” case.

The registration results of the “farthest” and the “closest” cases for ICP and ICP-PS were shown in Fig 6. When the number of points was beyond eight for both cases, ICP-PS provided more accurate registration results than standard ICP. In the “farthest” case, when the number of points was 25, the RMSE was 5.88 mm for ICP method and was 3.96 mm for ICP-PS, decreasing by 32.65%. In the “closest” case, ICP-PS achieved an RMSE of 2.81 mm relative to 5.84 mm for the ICP for the same 25 points, decreasing by 51.88%. The histograms of the UPLE of 25 points for two cases was shown in Fig 6c and 6d. Different from the simulation study, (where the theoretically maximal UPLE was set at 2 mm), the maximum UPLE of the “farthest” case was 4.25 mm and that of the “closest” case was 3.68 mm.

## Discussion

In this study, we compared our proposed ICP-PS method with standard ICP in both numerical simulation and cadaveric experiment. The simulation approach was used to assess the effects of two basic factors on the registration accuracy: (1) the number of registration points; (2) the level of UPLE. Based on simulation and experimental results, our method outperformed standard ICP registration. Our method provided an effective solution for registering a known shape model to a small number of points, which demonstrated the potential for A-mode US based intraoperative registration in upper and lower extremity orthopedic surgeries, e.g. skeletal positioning in TKA, THA and orthopedic surgeries in elbow and should joint. Furthermore, we have provided a framework for estimating the number of points for a required accuracy at a known level of error, which gives valuable information for experimental assessments in orthopedic surgery.

We found that, relative to the standard ICP method, our proposed registration method improved the accuracy of the final registration quite remarkably when the amplitude of UPLE was small, e.g. 0 to 1 mm (Fig 6). The Perturbation Search was able to avoid local minima encountered by the ICP. The inclusion of a perturbation search is thus a quite powerful method to minimize the PSD value and to reach a better solution. Compared to previously reported methods that randomly perturbed points [14,29], our method used prior knowledge concerning the geometrical constraints imposed to a shape model in order to get a more efficient search. The accuracy obtained in the simulation showed similar results to other studies [15, 20, 22, 31]. However, different accuracy metrics were used in various scenarios and made the comparison difficult. Commonly, an error of 1 mm Target Registration Error is acceptable. In our simulation, we found that 15 points would achieve a RMSE error below 1 mm when UPLE is zero.

The cadaver experiment results showed that the registration accuracy improved with an increasing number of surface sample points in general. However, this improvement highly depended on the error of each newly added point (see Fig 6c and 6d). When the maximal error of the registration points was 3.68 mm (Fig 6d), only 2.81 mm RMSE could be achieved by ICP-PS. Since the registration points were added based on descending order of UPLE, the points with the maximum UPLE for both cases always existed when the number of points increased from 6 to 25. The magnitudes of UPLE of all registration points influenced the final registration accuracy. The UPLE must be carefully reduced when the ICP-PS would be applied in the clinical practice. Although the UPLE of cadaver experiment was bigger than that of the numerical simulation, ICP-PS method still yielded a more accurate result than standard ICP. In this study, technical feasibility has been investigated. Clinical feasibility regarding to specific surgeries (e.g. TKA, THA) would be investigated in future study. Heger et. al. performed a similar study, where they also found that the addition of palpation points on the distal femoral region improved the registration accuracy[31]. However, they used a mechanical probe to touch the exposed bone surface for pre-registration, which resulted in a more accurate initial guess than US based pre-registration.

When the UPLE was zero, perfect registration (0 mm RMSE) was possible using ICP-PS in 80 out of 100 cases using 25 sample points. Standard ICP reached perfect registration in only 13 out of 100 cases for the same amount of sample points. Some cases could not reach the perfect registration result. Closer inspection of those cases revealed that the addition of random sample points can also lead to the inclusion of registration points with less contribution to the final result. For example, a newly added point may not anatomically be important or homogenously distributed (e.g. close to previously selected points) and that thus do not give adequately extra information for the registration. Thus, the spatial distribution of SSP must provide enough geometrical constraints for ICP-PS registration.

In this study we did not distinguish among sources of UPLE in the ultrasound data [20, 22] as all of these sources of error were difficult to quantify and measure in practice. In practice, the various sources of UPLE may have slightly different effects on the registration accuracy. To simplify the complexity of noise distribution in the simulation phase, we used an isotropic error model that a random direction vector that represented a noise signal was added to the SSP. In general, the registration accuracy increases by adding more points, but it will never be beyond a certain registration limit that is associated with the magnitude of UPLE. Hence, reducing the UPLE during measurement would provide an effective way to improve registration accuracy.

As we found in the numerical simulation and cadaver experiment, UPLE is a critical parameter that could affect the overall registration accuracy. However, the magnitude of UPLE is difficult to quantify in clinical practice. In some cases, a tracked mechanical sensor may produce a higher accuracy than a navigated A-mode US probe. Hence the proposed ICP-PS algorithm can also be applied for other digitalizing sensors. To facilitate the usage of A-mode US probe in CAOS, the improvement of localization accuracy of detected ultrasound point is a necessary step. A systematic investigation of A-mode US performance on special anatomical locations would provide valuable information to improve the localization accuracy, regarding to ultrasound waveform accessibility and intensity of received echo. The more robust and accurate calibration method should be developed in the future. An in-vivo experiment should be conducted to investigate the actual speed of sound in the future.

This study has some other limitations. Firstly, the numerical simulation we used differs from a real world situation. Nevertheless, we chose to use a simulation approach because that allowed us to assess the effects of individual parameters systematically without interference of other parameters such as additional experimental errors and other uncertainties that may occur in an experimental setup. The registration procedure as demonstrated with such a numerical simulation should always be validated in *in-vitro* situations before proceeding to *in-vivo* situations (surgery). Secondly, we only compared our proposed registration algorithm with standard ICP. Still, further research could extend the applicability of our proposed simulation method to other registration algorithms for other comparisons (e.g. Gaussian Mixture model registration[33]), which could also be tested in the simulation framework as described in this study. Thirdly, we used an empirically designed perturbation approach based on the observation of registration results to avoid local minima. Due to the object of our registration study being a femur, which is a long cylinder-like shape, most of the ‘looseness’ in 3D geometry is along the distal-proximal axis. For other shapes, the principle component analysis (PCA) can be applied on the known shape model to determine the perturbation axis. The principle of avoiding local minimum for other registration algorithms will be equally applicable for all situations where a point cloud is needed to register to a known shape model. More suitable perturbation approaches should be investigated and designed carefully based on different purposes.

## Conclusions

This study has presented a simulation approach to investigate the effects of the number of registration points and the Ultrasound Point Localization Error on the registration accuracy for intraoperative registration in CAOS. The simulation results were then verified in the cadaver experiment. A registration algorithm using less points to achieve a high accuracy was established and validated. The addition of a perturbation search and feedback to ICP resulted in significant improvement of accuracy compared to standard ICP. Furthermore, the simulation approach provides a well-defined framework for estimating the minimally required number of registration points for point cloud registration, once the required levels of accuracy and time efficiency have been set and the UPLE can be estimated. With the high potential to implement further improvements associated with the localization accuracy of acquired registration points, the proposed ICP-PS method would be suitable to accurately estimate bone position and orientation.
